# Valorization of Agro-Wastes as Fillers in PLA-Based Biocomposites for Increasing Sustainability in Fused Deposition Modeling Additive Manufacturing

**DOI:** 10.3390/ma17061421

**Published:** 2024-03-20

**Authors:** Niccolò Giani, Emanuele Maccaferri, Tiziana Benelli, Marco Bovo, Daniele Torreggiani, Enrico Gianfranco Campari, Patrizia Tassinari, Loris Giorgini, Laura Mazzocchetti

**Affiliations:** 1 Department of Industrial Chemistry “Toso Montanari”, University of Bologna, Viale Risorgimento 4, 40136 Bologna, Italy; niccolo.giani@unibo.it (N.G.); loris.giorgini@unibo.it (L.G.); laura.mazzocchetti@unibo.it (L.M.); 2 Interdepartmental Center for Industrial Research on Advanced Applications in Mechanical Engineering and Materials Technology, CIRI-MAM, University of Bologna, Viale Risorgimento 2, 40136 Bologna, Italy; 3 National Interuniversity Consortium of Materials Science and Technology (INSTM), 50121 Florence, Italy; enrico.campari@unibo.it; 4 Department of Agricultural and Food Sciences, Alma Mater Studiorum Università di Bologna, Viale G. Fanin 48, 40127 Bologna, Italy; marco.bovo@unibo.it (M.B.); daniele.torreggiani@unibo.it (D.T.); patrizia.tassinari@unibo.it (P.T.); 5 Department of Physic and Astronomy “Augusto Righi”, University of Bologna, Viale Carlo Berti Pichat 6/2, 40127 Bologna, Italy

**Keywords:** additive manufacturing, biocomposite, biofiller, poly(lactic acid)

## Abstract

The use of wheat middlings (WM) and rice husks (RH) as biofillers for mixing with poly(lactic acid) (PLA) matrix to produce new 3D-printable biocomposites was assessed. Filaments containing 10 and 20 wt.% agro-waste-derived biofillers were manufactured and, for the sake of comparison, filaments of neat PLA were also produced. The obtained filaments were characterized via thermogravimetric analysis (TGA) and differential scanning calorimetry (DSC), showing potential for further application in additive manufacturing processing. Three-dimensionally printed specimens were thus produced and characterized via: DSC, also evaluating the specific heat capacity (CP) of specific 3D-printed specimens; dynamic mechanical analysis (DMA), also applied for determining the coefficient of linear thermal expansion (CLTE) measured on 3D-printed specimens in two different directions (*X* and *Y*); and tensile tests. The latter testing campaign was carried out along three printing directions (*X*, *Y*, and *Z* axes) to test the intrinsic biocomposite features (*X*-printed samples) as well as interbead and interlayer adhesion (*Y*- and *Z*-printed specimens, respectively). All samples demonstrated acceptable properties. The inclusion of a cost-free natural material leads to a strong reduction of the whole material cost. Implementing this new class of composite material to an additive manufacturing technique can significantly reduce the environmental impact of 3D-printed products.

## 1. Introduction

Biobased polymers have been the subject of many studies over the last decade because of the increasing need to reduce the carbon footprint [1,2,3] of more traditional synthetic polymers. While biopolymers are often quite expansive with respect to traditional materials, their cost and potentially also their properties could be significantly improved upon employment of biobased fillers, and more precisely, agricultural wastes, in order to keep the carbon footprint limited, bringing several advantages, such as full circularity of resources, biodegradability, low specific gravity, and reduction of whole material cost [2]. In this regard, poly(lactic acid) (PLA) appears as an optimal candidate as a biocomposite matrix [4], owing to its large availability in comparison with other biobased thermoplastics, with 20.7% of the global production capacity in 2022 by material type [5], its full biodegradability, and the wide range of applications that could benefit from lowering of the material’s cost. Additionally, PLA is largely adopted in additive manufacturing (AM), in particular in fused deposition modeling (FDM), where it actually represents a benchmark for household applications [6]. Moreover, the feasibility of using PLA has already been demonstrated as a convenient matrix for producing reinforced 3D-printable filaments to obtain thermoplastic composites with enhanced characteristics [7]. In FDM processing, the approach of using PLA-based biocomposites has been proposed and discussed in the scientific literature, which has investigated a wide range of natural fillers, which, on the commercial scale, are mostly declined as wood/PLA filaments [8]. Several studies were focused on the development of biocomposite filaments using lignocellulosic fibers, such as wood [9], flax [10], bamboo [11], hemp [12], and kenaf [13]. Concurrently, a great part of the research tried to valorize residues from industry or agriculture, often produced locally, using them as biofillers in 3D-printable filaments, such as sugarcane bagasse [14] from sugar production, rice straw [15], or coconut wastes [16]. Indeed, FDM technology aims to take advantage of agricultural waste’s decreasing material cost [17] and environmental impact [18], which can also help reduce distortion after processing [19] while preserving the mechanical properties of the material [20]. Unfortunately, as reported in the literature, the majority of lignocellulosic biofillers provide materials with higher brittleness and lower tensile strength [13]; higher mechanical properties were reported mainly in the cases of flax [10], bamboo [11], and hemp fiber [12,19] biocomposites. Such behavior can be due to many reasons, such as: a large internal void content in the filament (intrabead voids) [13] because of the mixing process of heterogeneous materials; the well-known poor adhesion between biofillers and polymeric matrices, sometimes due to their different chemical natures, which does not allow for an efficient load transfer; phase segregation, which leads to inhomogeneous distribution and limited mechanical performance; and finally, high melt viscosity, which leads, in 3D printing, to low interlayer adhesion and high interbead void content. Some of these issues can be partially solved by surface treatments of biofillers and the use of compatibilizers.

In this context, we focused our interest on evaluating the possibility of using locally sourced agricultural wastes as biofillers for PLA in the production of polymeric filaments for FDM application. PLA was thus modified by the addition of wastes derived by the milling of wheat and rice, i.e., wheat middlings (WM) and rice husks (RH).

The wheat grain, i.e., wheat caryopsis, has a multi-layered structure. During the milling of wheat into flour, the starchy endosperm (precursor of edible flour) is separated from the bran material and embryo. The endosperm fraction is further ground while the bran material is a by-product, comprising different outer layers such as the embryo, aleurone layer, seed coat, and inner and outer pericarp layers [21,22]. These by-product fractions (called wheat middlings), however, do not have any specific applications and currently they are disposed of or sold at very low cost and mostly used for animal feed. Many studies dealing with the composition and nutritional value of wheat middlings [23,24,25] were reported, but, to the best of the authors’ knowledge, no one has investigated the possibility of using this residual fraction, rich both in protein and starch, for the production of polymeric composites. In this regard, the research group has already achieved a viable biocomposite processed as filaments for additive manufacturing FDM [26] with promising features. 

Another potential alternative among locally sourced agro-wastes is represented by rice husks, which are the protective hulls of the rice grains and represent the main by-product of rice milling. Indeed, it is reported that for every ton of milled rice, about 0.23 tons of rice husks are generated [27], and the global rice husk production is estimated to be between 128 and 148 million tons per year [28]. Amongst the wide range of available lignocellulosic feedstocks, rice husks have gained interest due to their aforementioned abundancy, and their elevated content of inorganic ash, approximately 20–25 wt.%. This inorganic fraction was extensively investigated, confirming that 90–95 wt.% is composed of amorphous silica [27,29]. Such a significant inorganic content makes rice husks abrasive and characterized by a low content of nutritious substances, which in turn make them unsuitable as food. Moreover, differently from a lot of other agricultural residues, they cannot be used as raw materials for the paper production industry or as an efficient natural fertilizer [28,29], owing to their moisture, oxygen, and soil humification resistance, and they is often burnt in air, releasing large amounts of hazardous substances into the atmosphere [25]. 

A preliminary evaluation of the ability to obtain a biocomposite filament suitable for 3D printing disclosed the versatility of WM as biofiller [26]. In this work, hence, the application of such a biocomposite for FDM will be discussed and evaluated. Moreover, a similar approach was addressed for RH as biofiller. The optimized compounding process was then used to produce new 3D-printable filaments containing 10 and 20 wt.% of WM and RH. The morphological aspect of RH was compared to that of WM in order to evaluate its suitability for the FDM technique. Thus, the second stage of the work was focused on the thermal and mechanical properties of 3D-printed biocomposites and the effect of different biofiller concentrations on them. Initially, all biocomposite filaments were studied in terms of thermal properties, via DSC and TGA, and then processed by FDM to obtain 3D-printed specimens. Among the thermal behaviors, *C_P_* and CLTE properties were studied. Thermomechanical and mechanical characterizations were carried out by means of DMA and tensile tests. Both CLTE measurements and tensile tests were conducted on two kinds of specimens with two distinct printing angles (0° and 90°) to assess the anisotropy of 3D-printed biocomposites.

## 2. Materials and Methods

### 2.1. Materials 

Poly(lactic acid) (PLA), with the commercial name IngeoTM 4043D (NatureWorks, Plymouth, MN, USA), was used as the matrix to produce composite filaments. It has a melt flow rate (MFR) of 6 g/10 min (210 °C, 2.16 kg). Flour waste (wheat middlings, WM), kindly provided by Molino Pivetti s.p.a. (Renazzo, FE, Italy), was composed of three fractions that differed in particle size, the finer of which was used after sieving with a 200 μm mesh sieve. The rice husks (RH) were supplied by local farm activity from Riseria Campanini S.R.L. (Ghisiolo, MN, Italy) and was subsequently milled for finer particle production.

### 2.2. Filament Production and 3D Printing

WM powder was sieved using a sieve with 200 µm mesh. RH biomass was milled by means of the Pulverisette 6 planetary ball mill (Fritsch, Idar-Oberstein, Germany) using a ZrO_2_ grinding jar and six ZrO_2_ balls, of which four were 10 mm in diameter and two were 20 mm in diameter. PLA pellets and biofillers were dried in oven at 75 °C for 1 h and kept in a vacuum bag before the first thermal compounding. The first extrusion was performed on the Process 11 twin-screw extruder (Thermo Fisher, Waltham, MA, USA) to efficiently mix the materials, applying the following processing conditions: temperature profile of the screw 175–180–180–185–185–185–170–150 °C; screw speed of 140 rpm. The process required a preliminary adjustment of the feeding screw for PLA pellets and biofillers, respectively set at 4 rpm and 2–4 rpm, to collect an extrudate composed of 10 and 20 wt.% biofillers. The extrudate materials were cooled by compressed air and then driven to a pelletizer. The pelletized material was dried again in oven at 75 °C for 1 h before the next extrusion. The second extrusion was performed on the NEXT 4.0 Advanced (3Devo, Utrecht, The Netherlands) single-screw extruder with four heating zones (165–170–175–165 °C) using a screw speed up to 5 rpm. This extruder was equipped with an optical sensor that allowed 3D-printable filaments with the desired diameter (1.75 mm) to be obtained.

Specimens for characterization tests were printed with a Mustang M400 3D printer (Vepram Vetoplast s.a.s., Castenaso, BO, Italy) at 230 °C, heated bed at 60 °C, 20 mm/s printing speed, 0.2 mm as layer height, 100% of infill, and using a nozzle diameter of 0.5 mm. To consider a possible variation in composition of the compounded filament, three repetitions were performed for each analysis. 

### 2.3. Characterization Methods

The evaluation of silica content in RH was performed by degradation test in a muffle oven. The RH powder was heated using a stepwise temperature ramp up to 600 °C in air. The inorganic residue was analyzed using the FTIR-ATR Alpha spectrometer (Bruker Alpha, Billerica, MA, USA) equipped with a diamond crystal. The analysis was performed by running 32 scans from 400 cm^−1^ to 4000 cm^−1^ with 4 cm^−1^ resolution.

The thermal stability of PLA and biocomposite filaments was investigated by thermogravimetric analysis (TGA) carried out on a TA Instrument SDT Q600 at 20 °C/min from room temperature to 600 °C under nitrogen flow (100 mL/min). The thermal degradation (*T_d_*) was defined as the onset extrapolation of the first relevant weight loss occurring during heating, while *W_r,N_*_2_ and *W_r,air_* refer to the solid residue, evaluated as weight fraction, retrieved at the end of the run at 600 °C in nitrogen or air atmosphere, respectively. 

Differential scanning calorimetry (DSC) measurements were performed on a TA Instrument Q2000 DSC modulated apparatus equipped with an RCS cooling system and calibrated with indium standards. Heating scans were run at 10 °C/min from 0 °C to 220 °C, in a nitrogen atmosphere. Between heating scans, a constant rate cooling (10 °C/min) was applied. Cold crystallization temperature (*T_cc_*) and melting temperature (*T_m_*) were taken at the peak maximum of the exotherm and endotherm, respectively. In the presence of multiple endotherms, the temperature of the most intense peak was taken as *T_m_*. The degree of crystallinity (*χ*) was evaluated considering the initial crystallinity of the material, calculated as the “total” melting enthalpy (Δ*H_m_*) minus the cold crystallization enthalpy (Δ*H_cc_*), and the theoretical melting enthalpy of 100% crystalline PLA (Δ*H_m,0_* = 93.7 J/g [30]), according to Equation (1):

(1) $$\chi \;(\%)=\frac{\left(\Delta H_{m}-{\Delta H}_{cc}\right)}{{\Delta H}_{m,0}}\cdot 100$$

Enthalpy values were normalized with respect to the actual PLA content in the biocomposites. DSC runs were carried out in triplicate for each sample on approximately 5 mg of filaments and 3D-printed materials. The Q2000 DSC modulated apparatus was also used for the determination of the specific heat capacity (CP) of thin 3D-printed specimens (2 layer height). The analysis was carried out by setting the heat flow at 30 °C equal to zero (target temperature for CP measurement), using a sampling interval of 1 pt/sec and heating the sample from 10 °C to 50 °C (heating rate of 10 °C/min). A calibration using a sapphire standard sample was required to assess the heat capacity of the instrument cell prior to each set of analyses. The analysis was carried out in triplicate for each formulated filament.

The dynamic mechanical analysis (DMA) of 3D-printed specimens was carried out using a DMA model 242E Artemis (Netzsch, Selb, Germany). Three bar-shaped (25 × 5 × 3 mm) specimens for each formulation were tested in tensile mode, heating from 30 °C to 100 °C (heating rate 3 °C/min) with 1 Hz oscillation frequency, 8.0 N dynamic force, 0.3 N constant static force, 1.1 proportional factor (PF, ratio of static force to dynamic force), and 50 µm strain. *E′_30 °C_* is the conservative modulus (*E′*) at 30 °C, *T_onset_* is the onset temperature of *E′* drop, and *tanδ* is the loss factor.

The coefficient of linear thermal expansion (CLTE) was measured using the above-mentioned DMA apparatus pre-set in TMA mode, in tensile deformation. After preliminary calibration with a standard steel specimen carried out under the same conditions as the CTE evaluation, printed specimens were tested applying a static force of 0.05 N during the analysis, heating from 30 to 100 °C at 2 °C/min. The same shape of DMA specimen (30 × 5 × 2 mm) was used. The probe displacement (dL) was recorded and CLTE was calculated as reported in Equation (2):

(2) $$CLTE\;\left(\alpha \right)=\frac{dL}{\Delta T}\cdot \frac{1}{L_{0}}$$

where *L*_0_ is the initial gauge length (about 10 mm, carefully defined for each specific specimen) following the main directives of ASTM E831-19 [31]. 

Tensile tests on 3D-printed dumbbell specimens were carried at RT according to ASTM 638 [32] Type V using a universal tensile testing machine (REMET TC10) equipped with a 10 kN load cell, under a crosshead speed of 1 mm/min. Three kinds of dogbone specimens were tested for each formulation: *X*-, *Y*- and *Z*-specimens with 5 replicates for each batch.

Optical microscopy of biofillers was carried out using a Hirox digital microscope with 50× magnification.

## 3. Results and Discussion

**Figure 1:**
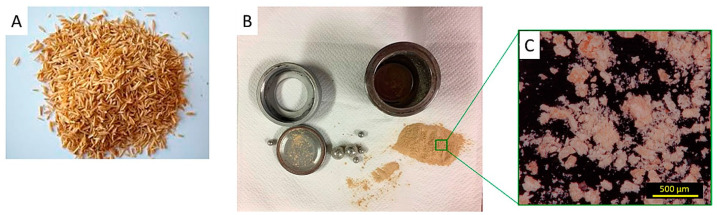
Rice husks (RH) pictured as received (**A**) and after planetary ball milling (**B**), also in micrographs obtained via optical microscopy (**C**), scale bar 500 μm.

Considering the Italian national wheat production of recent years, we can deduce that, every year in Italy, about 500,000 tons of wheat flour are produced. If we consider about 10% waste, there are about 450,000 tons of usable wheat middlings produced at a national level. The conservation of wheat meal and rice husks is not complex if the wastes are properly dried and then stored in a dry environment away from insects and fungi. So, the storage phase does not represent a complex problem. A preliminary evaluation of the feasibility of using 10% WM for 3D-printable filaments was reported by the authors, demonstrating the potential of such biofiller [26]. In that case, different fractions of wheat waste, with different coarseness, were considered (Figure S1 in Supplementary Information). The coarser ones, such as the so-called fine bran, comprising particles over 500 μm in size, were excluded, while the finer fraction, i.e., the middlings, was positively evaluated when used after sieving with a 200 μm mesh sieve [26]. Concerning RH, the starting particle size of the residue as gathered from the producer (Figure 1A) was reminiscent of the average dimension of the rice grain (a few mm up to more than a cm in length), which made this biomass unusable as biofiller in the 3D-printable filaments since the dimension is much higher than the nozzle diameter, i.e., 500 µm. Such a consideration imposed the downsizing of the biomass through planetary ball milling, reaching sub-millimeter dimensions (Figure 1B,C), which was in the order of a few hundred microns. In this case, sieving was also applied, though this ended up being pointless with the particles all passing through the mesh with only a negligible fraction separated.

As widely reported in the literature, RH is differentiated from other lignocellulosic materials due to its moisture absorption resistance [28]. This unique feature is conferred by its coating, mainly composed of silica. For this reason, a preliminary evaluation of the RH silica fraction within the biomass was carried out. Such a parameter has been reported to depend on crop region, weather, temperature, and nutritious substances, and the literature confirms that the silica amount ranges from 15 to 20 wt.% [27,28,29]. Hence, RH powder was heated in a muffle oven in an air atmosphere up to 600 °C to fully remove the organic fraction of the biomass. The process led to a final inorganic residue of 16 wt.%, identified as silica via FT-IR spectroscopy (See Figure S2 in Supplementary Information for the RH aspect before and after oxidation and the FT-IR spectrum of the residue), a fraction compatible with the ones reported in the literature [27,28,29].

An optimized processing method, made up of two subsequent extrusion steps, was used in order to obtain PLA-biocomposite filaments suitable for 3D printing (1.75 mm controlled diameter) containing 10 and 20 wt.% RH biofiller (Table 1), according to previously published results [26], as well as a PLA-biocomposite filaments containing 20 wt.% WM biofiller (Table 1) to be used for the production of 3D-printed specimens. Moreover, the previously obtained filament made of PLA with 10% WM [26] will be further discussed in comparison with the newly produced samples and finally used for 3D printing. In this context, considering the fraction of WM up to 20% wt in the biocomposite, theoretically about 2 million tons of biocomposite filament could be obtained every year only based on the Italian market. A comprehensive table of the biocomposites used in this work is reported in Table 1. 

**Figure 2:**
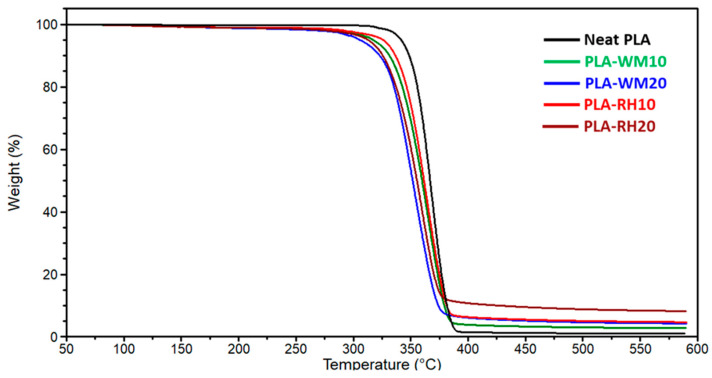
TGA thermograms (nitrogen atmosphere) of produced filaments: neat PLA (**—**), PLA-WM10 (**—**), PLA-WM20 (**—**), PLA-RH10 (**—**), and PLA-RH20 (**—**). Thermograms displayed in the figure were selected to best fit the average thermal behavior of each sample.

The thermal stability of the obtained biocomposite filaments was evaluated by TGA and compared with respect to PLA neat filament (Figure 2). The presence of biofillers was already proved to slightly reduce the thermal stability of biocomposites [26], and results from the present investigation confirmed (Table 2) such behavior. Indeed, *T_d_* values dropped from 352 °C of neat PLA filament down to about 330 °C for PLA-WM20 and PLA-RH20, with a stronger impact the higher the biofiller content. Similar results were reported for PLA-biocomposite containing natural fillers such as coconut waste [16] or kenaf fibers [33], and such a drop could be ascribed to the low thermal stability of lignocellulosic biofillers. Indeed, the principal constituents of biomass are lignin, hemicellulose, and cellulose, and their onset thermal decomposition temperatures (*T_d_*) are established around 160, 220, and 315 °C, respectively [34]. Nevertheless, such a decrease in thermal stability should not compromise the use of these materials in 3D printing applications since thermal degradation does not significantly proceed in conditions close to 3D printing processing temperatures (up to 220–230 °C).

Moreover, TGA showed that PLA-RH biocomposites had almost twice as much residue under nitrogen atmosphere as compared to PLA-WM biocomposites (Figure 2 and Table 2), probably caused by the high silica content of RH biofiller. This assumption was confirmed by the residues in air (*W_r,air_*), wherein only inorganic compounds were gathered after burning all of the organic fractions (Table 2), and values related to PLA-RH biocomposites were 4.5 times higher than those of analog formulations of PLA-WM biocomposites. Considering 16 wt.% silica content in RH powder, as previously discussed, the silica contents in biocomposite filaments containing 10 and 20 wt.% RH were expected to be 1.6 and 3.2 wt.%, respectively. Despite TGA characterizing only a very small amount of sample, in the order of a few mg, the obtained silica residues (Table 2) accurately matched the theoretical expected values. Such a result, along with the small scatter in the data from samples taken in regions very far away from each other in the filaments, confirmed that the employed compounding process led to good homogeneity of biocomposite filaments.

**Figure 3:**
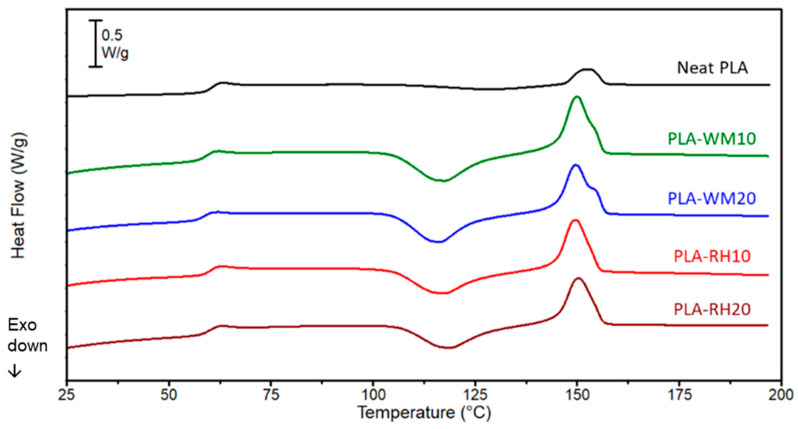
DSC thermograms (2nd heating scan) of neat PLA (**—**) and biocomposite filaments: PLA-WM10 (**—**), PLA-WM20 (**—**), PLA-RH10 (**—**), and PLA-RH20 (**—**). Thermograms displayed in the figure were selected to best fit the average thermal behavior of each sample.

The intrinsic thermal properties of all of the produced filaments were tested by DSC with two subsequent heating scans: in this context, the 2nd heating scan (Figure 3) was used, with the aim of first erasing the thermal history imparted by the filament extrusion process. As reported in Table 3, the thermal properties of PLA, i.e., the glass transition and melting temperatures, were preserved in the biocomposites after the addition of biofillers. Nevertheless, the nucleating effect of biofillers, already seen in the preliminary studies [26], led to higher enthalpies of cold crystallization (Δ*H_cc_*) and the lowering of the relative peak temperature, from 128 °C for neat PLA down to 115–118 °C for biocomposite filaments (Figure 3 and Table 3). However, the crystallinity of all biocomposites was close to zero, which represents an advantage for FDM applications, since highly crystalline filaments could provoke feeding troubles due to their excessive brittleness.

**Figure 4:**
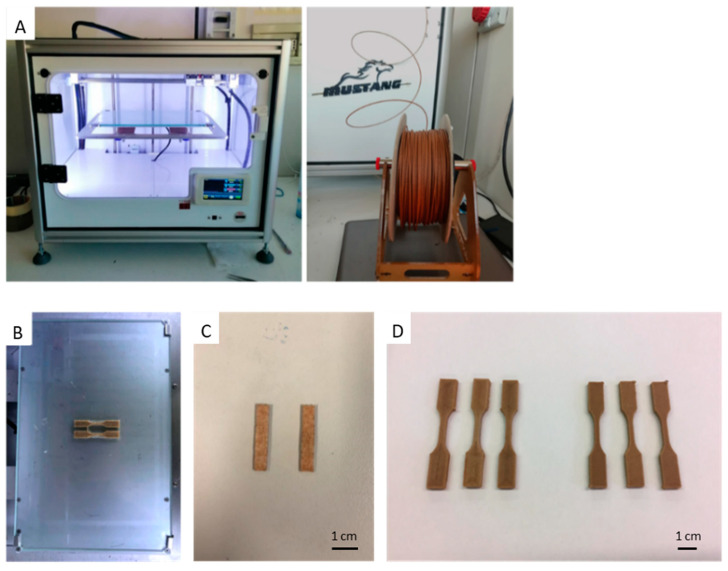
(**A**) Three-dimensional printer device used for the production of specimens. (**B**) Examples of 3D-printed dogbone specimens on the platform of the printer during production. (**C**) CLTE and DMA samples (right). (**D**) Dogbone samples to be used for stress/stain measurements.

The obtained filaments were used to manufacture 3D-printed specimens for mechanical and thermomechanical characterizations, using a commercial 3D printer (Figure 4A). In particular, bars were prepared for DMA and CLTE evaluation, instead, dogbone specimens (ASTM D638 Type V) were 3D printed to perform tensile tests (Figure 4B,D). Tensile tests were performed using three different specimens for each formulation with three specific printing orientations along the *X*-, *Y*- and *Z*-axes. The *X-* and *Y*-oriented specimens corresponded to 3D-printed samples with printing directions of 0° and 90° in the plane respectively, while *Z*-axis specimens corresponded to vertically 3D-printed dogbone specimens.

Very thin samples (with a 2 layer height) were also collected for the specific heat capacity (*C_P_*) measurements. Considering that *C_P_* accounts for 1 K of energy required for 1 °C of temperature increase for 1 g of material, its determination on a double-layered sample is useful to understand the impact of the layered structure on such a property, which, in turn, affects interlayer heat transfer during the printing process and can be responsible for the temperature gradient and inhomogeneous cooling within the printed object. All in all, no excessive deviations in *C_P_* value were detected in the studied composites. Since lignocellulosic materials are not typically highly thermally conductive [29,30,33,35,36], the addition of 10 wt.% both WM and RH provoked a slight increase in *C_P_* (Table 3). However, when the biofiller content was raised to 20 wt.%, an opposite trend in the two biomasses was recorded: while WM led to a fairly higher *C_P_* value, PLA-RH20 showed a lower *C_P_* than the same composite with just 10 wt.% RH, with an overall negligible variation along the series. In this regard, investigation of higher RH content would be interesting to assess whether a further reduction in *C_P_* would occur, possibly owing to the presence of silica in the filler, thus lowering the probability of incurring warping troubles.

**Figure 5:**
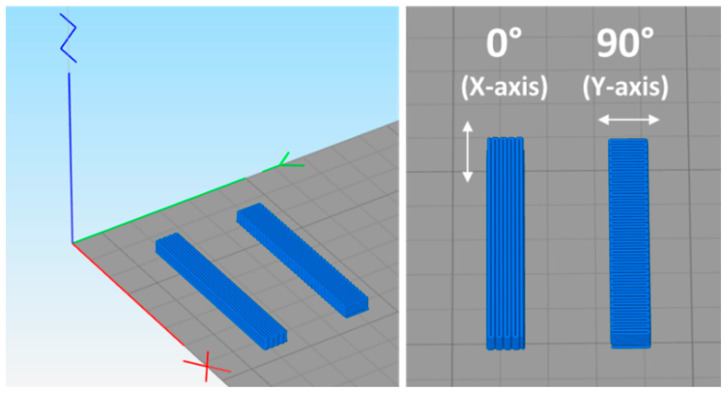
Slicing software view for the design of CLTE specimens with printing orientation along both *X*- (red) and *Y*-axes (green) of the building platform. CLTE measurements were carried out in both cases along the *X*-direction.

CLTE measurements are essential to assess the dimensional stability of 3D-printed samples and their tendency to warp during cooling. Indeed, a higher thermal expansion upon material heating entails a greater shrinkage upon cooling. In this context, CLTE values were calculated in the 10–50 °C range using samples produced with two filament deposition directions, namely 0° and 90° with respect to the assessment direction, as depicted in Figure 5.

**Figure 6:**
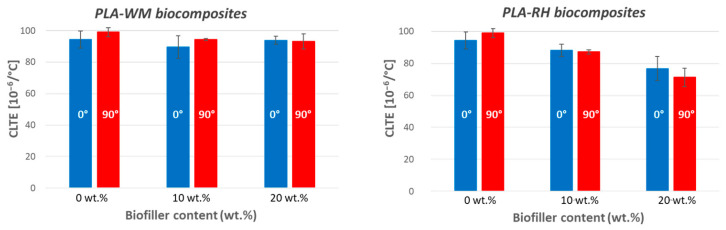
CLTE values as function of WM (**left**) and RH (**right**) content. Blue columns are relative to results of 0°-printed specimens and red columns for 90° ones.

As reported in the literature, in most cases, the addition of biofillers decreases the shrinkage effect during 3D printing due to macromolecular mobility inhibition [8]. An interesting point is that the 0° and 90° printing orientation results displayed a similar trend. The biocomposites with WM displayed only a slight decrease in CLTE, with a negligible effect of the filler content (Figure 6). On the other hand, when RH was used, decreasing CLTE values were recorded for both *X*- and *Y*-printed samples. This effect increased with increasing biofiller fraction. Such a different behavior when using WM or RH was already evidenced in the *C_P_* analysis. Such results could be ascribed to the presence of aggregates of biofillers, which formed at high WM concentration (20 wt.%). The resulting partial phase inhomogeneity did not allow for a strong reinforcing effect of WM toward PLA. The finer morphology of RH, instead, seemed to allow for a more homogeneous dispersion of the biofiller even when the RH concentration was 20 wt.%. In this way, RH biofiller positively impacted the thermal expansion of the biocomposite. Such results, along with the improved heat transfer properties reported in the *C_P_* analysis (Table 3), defined RH biomass as a promising biofiller for the prevention of some printing troubles, e.g., excessive shrinkage and warping.

**Figure 7:**
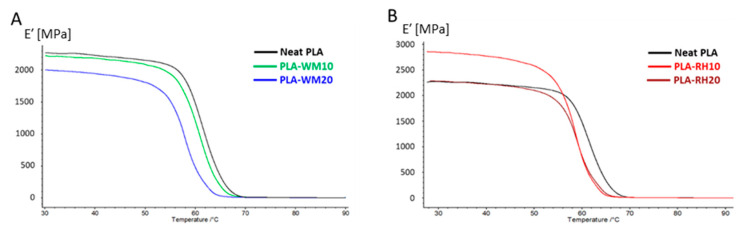
DMA spectra of: (**A**) PLA-WM biocomposites (PLA-WM10 **—** and PLA-WM20 **—**) and (**B**) PLA-RH biocomposites (PLA-RH10 **—** and PLA-RH20 **—**). Neat PLA curve (**—**) is also reported. Thermograms displayed in the figure were selected to best fit the average values calculated for each batch of specimens.

DMA in tensile mode was used to evaluate the conservative modulus (*E′*) of 3D-printed samples as a function of temperature. Specimens were 3D printed with a linear deposition pattern in the same direction as the applied force during the analysis (*X*-axis direction), since in CLTE measurements no significant differences in trends were observed between *X*- and *Y*-deposited samples. The DMA spectra of all samples showed a single dissipative phenomenon ascribed to the *T_g_* of PLA (Figure 7), after which the *E′* dropped to very low values since samples were left almost totally amorphous, as revealed by the DSC analysis (Table 3).

It is worth noting that the addition of 10 wt.% WM did not provide a significant effect on the storage modulus, giving results comparable to those of neat PLA (Table 4), whereas the addition of twice the WM amount (PLA-WM20) led to some decrease in the mechanical performance. Such a behavior was already tentatively hypothesized within the CLTE measurements, wherein 20 wt.% WM filling hinted at agglomerate formation. The effect of RH led to a similar behavior at the lowest load (PLA-RH10), where no significant mechanical improvement was detected, while on the other hand, 20 wt.% RH significantly boosted the conservative modulus of the biocomposite. 

**Figure 8:**
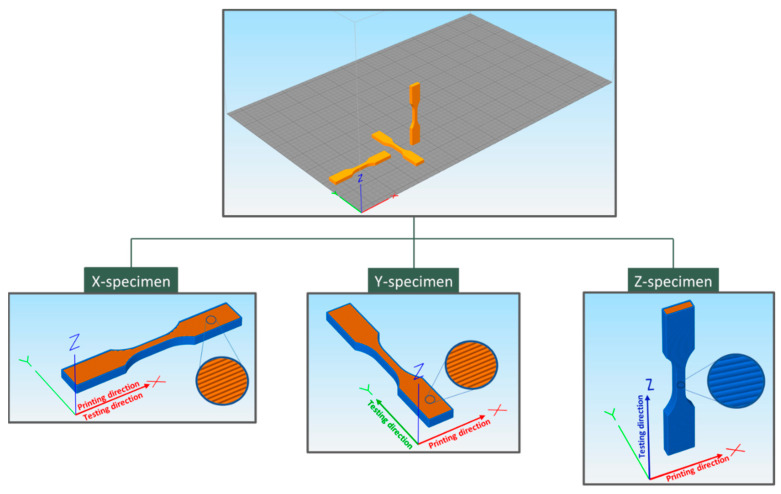
Slicing software view (Simplify3D) of dogbone specimens design used for the 3-dimensional mechanical characterization: *X*-specimen (**left**), *Y*-specimen (**middle**), and *Z*-specimen (**right**).

**Figure 9:**
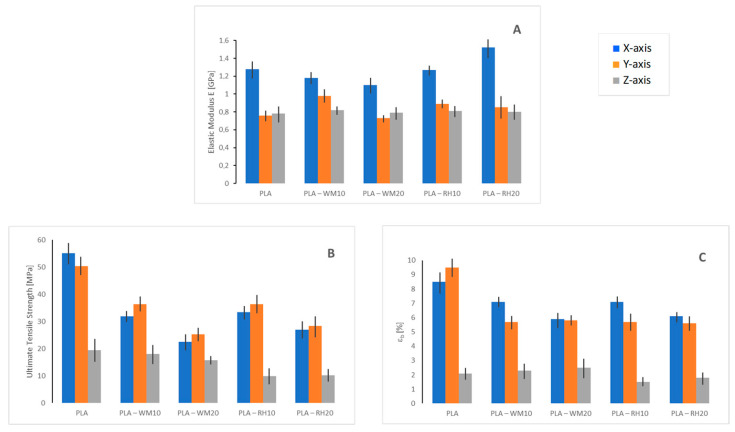
Tensile test results (X tensile stress applied) of samples 3D-printed along *X*-(∎), *Y*-(∎), and *Z*-axis (∎) directions: (**A**) elastic modulus; (**B**) ultimate tensile strength; and (**C**) elongation at break (*ε_break_*).

Finally, the mechanical properties were investigated by tensile tests using three different classes of specimens designed with the slicing software (Simplify3D V 5.0) and produced by growing the dogbone specimens along the three axes of the building platform (Figure 8) with 100% infill. In this regard, no actual perimeter was applied in the necked section of the dogbone to avoid influences on the stress/strain measurements. Hence, the printing direction was maintained on the *X*-axis for all specimens, while the testing direction varied according to the building orientation of the specimens, namely the *X*-, *Y*- and *Z*-axes, as depicted in Figure 8. During the deposition, the filament orientation was inverted at each layer by 180°. As *X*-axis specimens were made of extruded filaments (beads) parallelly disposed to the testing direction, *X*-specimens were helpful to evaluate the tensile properties of biocomposites; *Y*- and *Z*-specimens, instead, were produced for evaluating the extent of the interbead and interlayer adhesion, respectively. Indeed, *Y*-specimens were tested on the in-plane orthogonal direction (*Y*-axis) with respect to the bead deposition (*X*-axis). In this regard, we referred to the “bead” as the main filament section after deposition. Such a feature implied the load application on the interbead surface, and, consequently, the maximal stress achieved during the tensile test outlined the interbead adhesion. Similarly, *Z*-specimens were vertically manufactured and thus their characterization involved the load application on the orthogonal axis to the *X–Y* plane. In this way, interfaces between layers were forced and the ultimate strength reached during the analysis defined the interlayer strength or adhesion. All of the evaluated mechanical properties are summarized in Figure 9.

Considering the in-plane mechanical properties of 3D-printed biocomposites (*X*- and *Y*-specimens), the addition of WM led to a slight decrease in the elastic modulus with a concurrent drop in UTS and embrittlement with smaller elongation at break (*ε_break_*). These observations substantially confirmed the previously discussed data, wherein wheat middlings seemed to hardly homogenize with PLA, with a possible lack of adhesion between the biofiller and matrix that did not allow for proper load transfer between the two phases. Such an observation suggests that a surface treatment would probably be required to make the biofiller compatible with the thermoplastic matrix. RH, instead, led to some better moduli, strengthening the material, in particular when the *X*-printed sample was analyzed. Indeed, DMA tests were run in the very same specimen configuration. Concurring with the WM results, however, and as expected in the presence of a filler, both UTS and *ε_break_* dropped, with a behavior similar to WM-modified samples. All test results of *Y*-specimen biocomposites showed reductions in *σ_max_* and *ε_break_* with biofiller content, thus indicating a decrease in the interbead strength. Such a behavior seemed to suggest that the presence of biofillers hampered the macromolecular diffusion across the bead interface, thus leading to lower bead welding during 3D printing. 

The interlayer adhesion was evaluated by tensile tests on *Z*-specimens. As shown in Figure 9, all of the samples, even neat PLA, displayed overall lower mechanical properties with respect to the in-plane specimens (*X*- and *Y*-printed), while only the elastic modulus was reported to be similar to that of *Y*-printed samples. These characteristics suggested the *Z*-axis being the weak direction of the 3D-printed part. Such a behavior is already reported in literature [37], ascribed to the lowered diffusion of polymeric chains caused by the presence of a heterogeneous agent onto the layer–layer interface, and was already partially highlighted in the *Y*-direction (see similarities in the elastic modulus). This phenomenon was more evident with RH addition, which led to a more significant drop in ultimate stress, namely the interlayer strength, with respect to neat PLA (Figure 9B). WM biocomposites, instead, displayed interlayer strength values fairly close to the unfilled material. As a final result, the time in which the superficial section of the layer was in the molten state was fairly short, thus not achieving the optimal interlayer diffusion of macromolecules.

In terms of absolute performance, it is not an easy task to compare such results with other corresponding products owing to the limited literature and the fact that agro-wastes can be profoundly different and often details on their characterization and even simply dimensions are lacking. As a matter of fact, the use of agro-wastes, without any further modification, is significantly affected by the quality, type, and region of sourcing of the wastes themselves. A recent review paper [38] put forward a systematic approach for being able to compare many different products in AM starting from agro-wastes. Even in the review, only 57 papers were considered encompassing all of the areas of biomasses (fisheries, forests, as well as agriculture). Concerning wheat middlings, no reference other than the authors’ previously published note was considered, while for rice residues, only rice straw or rice husks isolated fibers were used, but with polypropylene as a polymeric matrix. The presently obtained results, however, encourage the future investigation of such an approach, possibly suggesting superficial treatment of biofillers or the employment of compatibilizers in order to enhance the filler/matrix adhesion and reach the maximum attainable mechanical properties.

A preliminary market survey shows, nonetheless, that wheat middlings and rice husks are currently listed on the Italian national market at a few Euro cents per kilo, i.e., 0.20–0.30 €/kg [39]. From an economic analysis, the authors determined that the cost of the operations necessary to select and mill these natural materials and to extrude filaments of biocomposite PLA-organic material can be obtained in a range from 3 to 5 € per kilo of extruded filament. Thus, the reduction of the cost of the whole material for printing, for example for filaments containing 20 wt.% WM, could be estimated in a range from 15% to 17%, with respect to the cost of neat PLA.

## 4. Conclusions

The present results demonstrate the possibility of producing more sustainable and cost-effective 3D printing materials by employing agricultural wastes, i.e., wheat middlings (WM) and rice husks (RH), as biofillers for thermoplastic filaments. For this purpose, filaments containing 10 and 20 wt.% WM and RH were successfully produced with an optimized and validated compounding process that did not provoke degradation of the PLA matrix. The investigation of the biocomposite filaments revealed good biofiller dispersion and convenient printability of all formulations, assuming the grain dimension was taken well below the nozzle diameter. Thermal characterization of the 3D-printed materials confirmed the suitability of the investigated biocomposites. Particularly, RH-composites showed some extent of decreasing both C_P_ and CLTE, with a linear dependence on the RH content, possibly due to the high silica content in the biomass, which suggests the positive effect of RH for shrinkage and warping prevention. Mechanical characterization revealed the progressive embrittlement of the biocomposites, while RH positively impacted the tensile elastic modulus of the biocomposites; however, the interlayer adhesion underwent a significant drop in the presence of RH as biofiller, outlining the hindering effect of RH on the macromolecular mobility. PLA-WM biocomposites, instead, seemed to suffer a scarce affinity towards PLA, thus suggesting that some surface modification could be required to boost biocomposite performance. The obtained results, anyway, are particularly intriguing considering the ease of fabrication of the biocomposite filaments, which are nonetheless always suitable for successful FDM application.

## Figures and Tables

**Table 1:** 3D-printable filaments used for specimen productions.

Filament	Biofiller	wt.% Biofiller
Neat PLA	-	-
PLA-WM10 [26]	WM	10
PLA-WM20	WM	20
PLA-RH10	RH	10
PLA-RH20	RH	20

**Table 2:** TGA results of reference PLA and biocomposite materials.

Sample	*T_d_* (°C)	*W_r,N_*_2_ (wt.%)	*W_r,air_* (wt.%)
PLA	352 ± 1	1.0 ± 0.1	<0.1
PLA-WM10	339 ± 3	2.7 ± 0.1	0.4 ± 0.1
PLA-WM20	329 ± 1	4.4 ± 0.1	0.7 ± 0.1
PLA-RH10	340 ± 2	5.0 ± 0.2	1.8 ± 0.2
PLA-RH20	332 ± 1	8.3 ± 0.4	3.2 ± 0.2

**Table 3:** Thermal properties from DSC measurements of PLA and biocomposite filaments.

Sample	*T_g_* [°C]	*T_cc_* [°C]	Δ*H_cc_* ^(a)^ [J/g]	*T_m_* [°C]	Δ*H_m_* ^(a)^ [J/g]	*χ* [%]	*C_P_* [J·°C^−1^·g^−1^]
Neat PLA	59	128	26.1 ± 0.8	151	27.4 ± 0.4	1.4	1.36 ± 0.07
PLA-WM10	59	117	33.6 ± 0.6	150	34.6 ± 0.4	1.0	1.41 ± 0.10
PLA-WM20	58	115	42.3 ± 1.8	150	43.4 ± 2.3	1.0	1.54 ± 0.05
PLA-RH10	59	117	29.6 ± 0.4	150	30.6 ± 0.4	1.0	1.38 ± 0.07
PLA-RH20	59	118	32.9 ± 1.6	150	35.5 ± 0.9	1.1	1.32 ± 0.02

(a) Enthalpy values were normalized with respect to the actual PLA content in the biocomposites.

**Table 4:** DMA results of neat PLA and biocomposites.

Sample	*E′*_30 °C_ (GPa)	*T_onset, E′_* (°C)
Neat PLA	2.3 ± 0.1	57 ± 1
PLA-WM10	2.3 ± 0.1	57 ± 1
PLA-WM20	2.0 ± 0.1	55 ± 1
PLA-RH10	2.4 ± 0.1	55 ± 1
PLA-RH20	2.8 ± 0.2	56 ± 1

## Data Availability

Dataset available upon request from the authors (due to privacy).

## Supplementary Materials

The following supporting information can be downloaded at: https://www.mdpi.com/article/10.3390/ma17061421/s1, Figure S1: Different particles in the different fractions of wheat middlings; Figure S2: Photographs of rice husks before and after removal of the organic fraction, together with the FT-IR spectrum of the white solid residee, which is typical of silica.
