# Supplementary material for: Valorization of Agro-Wastes as Fillers in PLA-Based Biocomposites for Increasing Sustainability in Fused Deposition Modeling Additive Manufacturing
Source: Materials (Basel). 2024 Mar 20;17(6):1421. doi: 10.3390/ma17061421 (PMC10971891; doi:10.3390/ma17061421)
Supplement: Supplementary file 1 [file materials-17-01421-s001.zip › materials-2889946-supplementary.pdf]

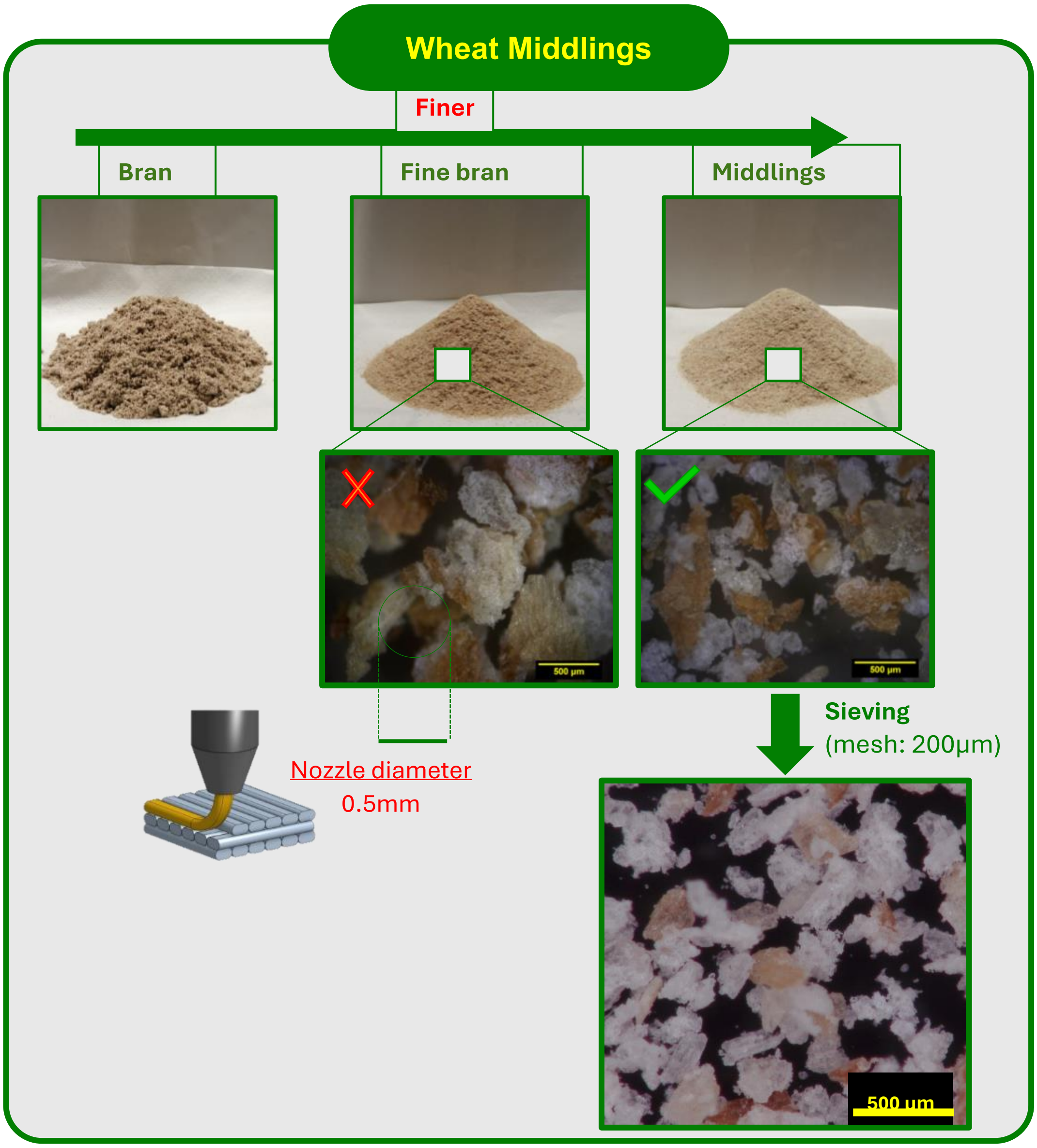

Figure S1. Different particles in the different fractions of wheat middlings.

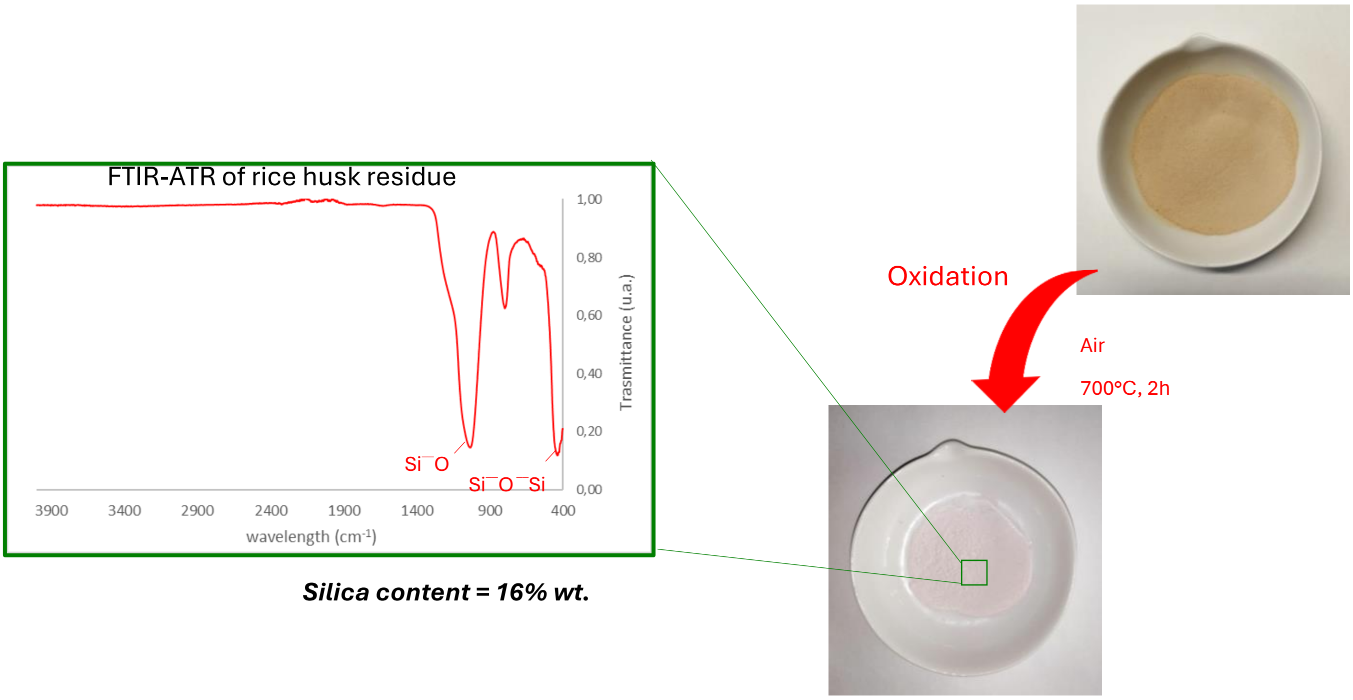

Figure S2. Photographs of the Rice Husk before and after removal of the organic fraction, toghether with the FT-IR Spectrum of the white solid resude which is typical of Silica.
